# Supplementary material for: The PROMISES study: a mixed methods approach to explore the acceptability of salivary progesterone testing for preterm birth risk among pregnant women and trained frontline healthcare workers in rural India
Source: BMJ Open. 2021 Jan 8;11(1):e040268. doi: 10.1136/bmjopen-2020-040268 (PMC7798670; doi:10.1136/bmjopen-2020-040268)
Supplement: Author's manuscript [file bmjopen-2020-040268.draft_revisions.pdf]

BMJ Open

BMJ Open is committed to open peer review. As part of this commitment we make the peer review history of every article we publish publicly available.

When an article is published we post the peer reviewers' comments and the authors' responses online. We also post the versions of the paper that were used during peer review. These are the versions that the peer review comments apply to.

The versions of the paper that follow are the versions that were submitted during the peer review process. They are not the versions of record or the final published versions. They should not be cited or distributed as the published version of this manuscript.

BMJ Open is an open access journal and the full, final, typeset and author-corrected version of record of the manuscript is available on our site with no access controls, subscription charges or pay-per-view fees (http://bmjopen.bmj.com).

If you have any questions on BMJ Open's open peer review process please email info.bmjopen@bmj.com

BMJ Open

The PROMISES Study: Impact of training, research involvement, and salivary progesterone testing on women and frontline health workers' understanding of preterm birth.

Journal:	*BMJ Open*
Manuscript ID	bmjopen-2020-040268
Article Type:	Original research
Date Submitted by the Author:	13-May-2020

Complete List of Authors:	Ashworth, Danielle; King's College London, Department of Women & Children's Health<br>Sharma, Pankhuri; University of Delhi, Department of Anthropology<br>Silverio, Sergio; King's College London, Department for Women & Children's Health<br>Khan, Simi; Deutsche Gesellschaft für Internationale Zusammenarbeit GmbH India Office, Indian Ministry of Food<br>Kathuri, Nishtha; MAMTA Health Institute for Mother and Child, Research and Innovation Unit<br>Garg, Priyanka; MAMTA Health Institute for Mother and Child, Research and Innovation Unit<br>Ghule, Mohan; MAMTA Health Institute for Mother and Child, Research and Innovation Unit<br>Shivkumar, V; Mahatma Gandhi Institute of Medical Sciences, Department of Pathology<br>Tayade, Atul; Mahatma Gandhi Institute of Medical Sciences, Department of Radio-Diagnosis<br>Mehra, Sunil; MAMTA Health Institute for Mother and Child, Young People and Sexual and Reproductive Health and Rights Unit<br>Shivkumar, Poonam; Mahatma Gandhi Institute of Medical Sciences, Department of Obstetrics and Gynecology<br>Tribe, Rachel M.; King's College London, Department of Women & Children's Health

Keywords:	PUBLIC HEALTH, Maternal medicine < OBSTETRICS, Prenatal diagnosis < OBSTETRICS

SCHOLARONE™
Manuscripts

I, the Submitting Author has the right to grant and does grant on behalf of all authors of the Work (as defined in the below author licence), an exclusive licence and/or a non-exclusive licence for contributions from authors who are: i) UK Crown employees; ii) where BMJ has agreed a CC-BY licence shall apply, and/or iii) in accordance with the terms applicable for US Federal Government officers or employees acting as part of their official duties; on a worldwide, perpetual, irrevocable, royalty-free basis to BMJ Publishing Group Ltd ("BMJ") its licensees and where the relevant Journal is co-owned by BMJ to the co-owners of the Journal, to publish the Work in this journal and any other BMJ products and to exploit all rights, as set out in our licence.

The Submitting Author accepts and understands that any supply made under these terms is made by BMJ to the Submitting Author unless you are acting as an employee on behalf of your employer or a postgraduate student of an affiliated institution which is paying any applicable article publishing charge ("APC") for Open Access articles. Where the Submitting Author wishes to make the Work available on an Open Access basis (and intends to pay the relevant APC), the terms of reuse of such Open Access shall be governed by a Creative Commons licence – details of these licences and which Creative Commons licence will apply to this Work are set out in our licence referred to above.

Other than as permitted in any relevant BMJ Author's Self Archiving Policies, I confirm this Work has not been accepted for publication elsewhere, is not being considered for publication elsewhere and does not duplicate material already published. I confirm all authors consent to publication of this Work and authorise the granting of this licence.

For peer review only

The PROMISES Study: Impact of training, research involvement, and salivary progesterone testing on women and frontline health workers' understanding of preterm birth.

Danielle C. Ashworth,[1‡]

Pankhuri Sharma,[2‡]

Sergio A. Silverio,[1] ORCiD: 0000-0001-7177-3471

Simi Khan,[3]

Nishtha Kathuria,[4]

Priyanka R. Garg,[4]

Mohan D. Ghule,[4]

V.B. Shivkumar,[5]

Atul Tayade,[6]

Sunil Mehra,[7]

Poonam V. Shivkumar,[8]

Rachel M. Tribe (rachel.tribe@kcl.ac.uk),[1±] ORCiD: 0000-0003-3675-9978

and on behalf of the PROMISES Study Team.*

‡ Joint 1st Authors

± Corresponding Author: Professor Rachel Tribe, Dept. of Women and Children's Health, School of Life Course Sciences, King's College London, St Thomas' Hospital, London, SE1 7EH, United Kingdom, rachel.tribe@kcl.ac.uk, 0207 188 8241 (direct), 0207 188 3639 (admin)

*** The wider PROMISES Study Team includes:** Paul T. Seed (Department of Women & Children's Health, King's College London); Archana Sarkar (Obstetrics and Gynecology, MAMTA Health Institute for Mother and Child); and Ritu Dargan, Lalita Sengupta, Vikram Singh (Research and Innovation Unit, MAMTA Health Institute for Mother and Child).

For peer review only

[1] Department of Women & Children's Health, King's College London, London, UK
[2] Department of Anthropology, University of Delhi, Delhi, India
[3] Indian Ministry of Food, Deutsche Gesellschaft für Internationale Zusammenarbeit, New Delhi, India
[4] Research and Innovation Unit, MAMTA Health Institute for Mother and Child, New Delhi, India
[5] Department of Pathology, Mahatma Gandhi Institute for Medical Sciences, Maharashtra, India
[6] Department of Radio-Diagnosis, Mahatma Gandhi Institute for Medical Sciences, Maharashtra, India
[7] Young People and Sexual and Reproductive Health and Rights Unit, MAMTA Health Institute for Mother and Child, New Delhi, India
[8] Department of Obstetrics and Gynecology, Mahatma Gandhi Institute for Medical Sciences, Maharashtra, India

Keywords: Preterm Birth, India, Salivary Progesterone, LMIC, Pregnancy

Word Count: 3,676

For peer review only

DECLARATIONS

Ethics approval and consent to participate

Ethical approval was obtained from both MAMTA Health Institute for Mother and Child (MAMTAHIMC) Ethical Review Board (MERB/Dec-2016/002) and the Institutional Review Board of Mahatma Gandhi Institute of Medical Science (MGIMS/IEC/OBGY/289/2016) prior to any recruitment. Written informed consent was obtained from pregnant women and ASHAs.

Consent for publication

Not applicable.

Data sharing statement

Data is not available to be shared.

For peer review only

Funding

This study was conducted as a part of project titled 'Low-cost salivary progesterone testing for detecting the risk of preterm births in rural community settings of India'. Funding for this project was provided by the Grand Challenge India, 2015 – All Children Thriving – India partners (Biotechnology Industry Research Assistance Council; Department of Biotechnology, India; and the Bill and Melinda Gates Foundation) for its overall implementation.

Authors' contributions

DCA, PS, SAS and RMT drafted the manuscript. NK made substantial contributions when reviewing and revising the manuscript. VBS, AS, PVS, PK, SK, RMT, SM, MDG and wider the PROMISES Study Team contributed to the conception and design of the PROMISES study.

Acknowledgements

We would like to thank all the women who took part in this study, the ASHAs, and the clinical staff who performed the ultrasound scans.

Competing Interests

The authors declare that they have no competing interests.

Disclosure statement

Sergio A. Silverio (King's College London) is supported by the National Institute for Health Research Applied Research Collaboration South London [NIHR ARC South London] at King's College Hospital NHS Foundation Trust. The views expressed are those of the authors and not necessarily those of the NIHR or the Department of Health and Social Care.

Patient and Public Involvement

We did not directly include PPI, but the study rationale was presented and discussed with our UK NIHR recognised Preterm birth PPI group and the acceptability of the research was informed by participant feedback from a similar UK based study. Due to the participatory nature of this research, this study provides important PPI from women and community healthcare workers in rural India who are rarely consulted about research.

For peer review only

ABSTRACT

Introduction: India has an overall neonatal mortality rate of 28/1000 live births, with higher rates in rural India. Approximately 3.6 million pregnancies in India are affected by preterm birth annually. The deaths associated with preterm birth in India contribute a quarter of the global preterm related deaths. Embedded within the PROMISES study (which aims to validate a low-cost salivary progesterone test for early detection of preterm birth risk) we present a mixed methods explanatory sequential feasibility sub-study of the salivary progesterone test.

Methods: A pre- and post-training questionnaire to assess Accredited Social Health Activists (n=201) knowledge and experience of preterm birth and salivary progesterone sampling was analysed using the McNemar test. Descriptive statistics for a cross-sectional survey of pregnant women (n=400) are presented in which the acceptability of this test for pregnant women is assessed. Structured interviews were undertaken with Accredited Social Health Activists (n=10) and pregnant women (n=9) and were analysed using Thematic Framework Analysis to explore the barriers and facilitators influencing the use of this test in rural India.

Results: Before training ASHAs knowledge of PTB (including risk factors, causes, postnatal support and testing) was very limited. After the training programme, there was a significant improvement in the ASHAs' knowledge of PTB. All four hundred women reported the salivary test was acceptable with the majority finding it easy but not quick or better than drawing blood. For the qualitative aspects of the study analysis of interview data with ASHAs and women, our thematic framework comprised of three main areas: Implementation of Intervention; Networks of Influence; and Access to Healthcare. Qualitative data were stratified and presented as barriers and facilitators.

Conclusion: This study suggests support for ongoing investigations validating preterm birth testing using salivary progesterone in rural settings.

For peer review only

ARTICLE SUMMARY: STRENGTHS AND LIMITATIONS OF THIS STUDY

- This is the first study to present a mixed methods approach to understanding the acceptability of salivary progesterone testing for risk of preterm birth in rural India.
- This study presents data supporting the introduction of a previously un-used point of care test which can easily and quickly be utilised outside of formal healthcare settings, and delivered by community healthcare workers.
- This study presents and integrates mixed methods data from both women and community healthcare workers in a rigorous and methodical way.
- This study was conducted in a low resource setting and demonstrates how a simple intervention can provide the possibility to improve the prenatal care of women and their babies.

KEY MESSAGES:

What is already known?

- Preterm birth is a common cause of neonatal death.
- India has one of the highest rates of neonatal mortality in the world.
- Salivary progesterone testing in pregnant women has proved promising for the early detection of risk of preterm birth.

What are the new findings?

- Knowledge of preterm birth and associated risks is generally low amongst women and community healthcare workers in rural India, despite it being a common occurrence.
- Salivary progesterone testing can be delivered effectively by community healthcare workers, and is deemed acceptable by both them and pregnant women.
- Barriers to accessing maternity care have been highlighted, whilst facilitators have been emphasised.

What do the new findings imply?

For peer review only

- Community healthcare workers are an efficient means to increase maternity care monitoring in rural settings.
- Salivary progesterone testing for risk of preterm birth in pregnant women is feasible and so should be pursued in future research and healthcare delivery.
- Maternity care in rural India is heavily influenced by a number of a number of social, familial, and lifestyle factors.

For peer review only

INTRODUCTION

Globally, India's contribution to neonatal mortality is highest in the world with an estimate of 0.75 million (Liu, Oza et al. 2015) neonatal deaths in 2013. India's neonatal mortality rate is 28 per thousand live births, and in rural India the situation is even graver with 31 neonatal deaths per thousand live births (Ministry of Home Affairs 2013). Preterm birth (PTB) has been identified as one of the significant causes of neonatal deaths both in world (Liu, Johnson et al. 2012) and in India (Liu, Oza et al. 2015). Approximately 3.6 million pregnancies in India are affected by PTB annually, of these, 300,000 (World Health Organization; March of Dimes; The Partnership for Maternal 2012) die and many surviving infants live with disability. Globally, PTBs and associated deaths are the highest in India, contributing to a quarter of the global preterm related deaths (World Health Organization; March of Dimes; The Partnership for Maternal 2012). This burden is further affected by the low awareness level and utilization of health services, an issue that is exacerbated in rural India. Knowledge of, and around, PTB is limited even among community level health workers. This lack of knowledge hinders identification of mothers at risk of PTB and can delay access to health care services to manage such pregnancies. Considering the high burden of preterm birth and lack of knowledge thereof, an innovative study with an aim to validate a low-cost salivary progesterone (PROMISES) is being conducted for early detection of risk of PTB among pregnant women in two rural districts of India (Sharma, Khan et al. 2018).

Even today, many myths and misconceptions prevail about pregnancy and childbirth in rural areas, mainly due to inadequate formal education, poor accessibility to healthcare services and lack of trained frontline health workers. Moreover, major role of decision making about health care of expectant mothers is mainly done by their immediate family members. The recent national survey in India indicates that only 11.4% women in rural India alone makes decision about their health care (NFHS-4). These contextual factors play a major role in implementing effective innovative interventions for improving maternal and neonatal health.

In rural India, government instituted community health care workers, Accredited Social Health Activist (ASHAs), play a significant role in linking pregnant women to maternal

For peer review only

and child health care services (Ministry of Health & Family Welfare 2017-2018). These frontline health workers work in their residential communities/villages as health activists, educators and providers of basic essential services. ASHAs provide several support services to pregnant women (Vellakkal, Reddy et al. 2017) and invest considerable effort in identifying expectant and building trust with expectant mothers and their family members. A multitude of evidence (Fathima, Raju et al. 2015, Tripathy, Nair et al. 2016) suggests that these links with frontline health workers have improved maternal and neonatal health interventions. For ASHA workers, the required minimum formal education (Ministry of Health & Family Welfare 2013) is only up to class 8th, hence it is important to provide adequate knowledge and regular trainings to ASHAs on topics of maternal and neonatal health in order to improve practice and sustainability of innovative interventions in these fields.

Hence, as part of the PROMISES study (Sharma, Khan et al. 2018) two of the aims were: a) to assess the knowledge and educate the frontline health workers, ASHAs, on PTB and salivary progesterone sampling through a training programme; and b) to consider the feasibility, and acceptability to women and health workers, of using salivary progesterone in the rural settings for its further scale up as 'point of care test'. More specifically, the research objectives were to determine:

1. If training frontline health workers improved their knowledge on PTB and salivary progesterone sampling
2. Whether salivary progesterone PTB tests were acceptable to frontline health workers and pregnant women
3. The range of facilitating factors and barriers influencing the use of salivary progesterone PTB tests in Indian rural settings

METHODS

STUDY DESIGN & SETTING

A mixed methods study was undertaken to explore knowledge of PTB in frontline healthcare workers, and the acceptability and feasibility of salivary progesterone testing through a survey and structured interviews, respectively. This work formed part

of a prospective study evaluating the feasibility and accuracy of saliva progesterone test to predict preterm birth, entitled – 'Low-cost salivary progesterone testing for detecting the risk of preterm births in rural community settings of India, PROMISES study' (Sharma, Khan et al. 2018). Participating healthcare workers were government instituted community health care workers, ASHAs, whose primary role is health education and promotion of good health practice and health service accessibility and utilization. ASHAs were instrumental in recruitment of pregnant women for the PROMISES study, and thus capacity building through training was essential for effective implementation. Furthermore, the study explored the acceptability of salivary progesterone test among pregnant women eligible for PROMISES.

We conducted a pre-post training assessment of ASHAs knowledge of PTB; structured interviews with a sample of pregnant women and ASHAs discussing acceptability and feasibility of salivary progesterone testing for PTB; and a cross-sectional survey of pregnant women recruited to PROMISES which included questions on acceptability of salivary progesterone testing.

This study was carried out in the state of Madhya Pradesh, India, within two districts Panna and Satna, selected because of their high fertility and neonatal mortality rates (The Lancet 2012, Ministry of Home Affairs 2014). A one-day training programme was conducted for ASHAs at eight primary health care centres (PHCs). Overall four one-day training programmes were conducted, three at primary health centres and one at a district field office. Training included information about the maternal and child health services within the health systems, outreach services, high risk pregnancies, newborn care, PTBs, and general diagnostic services in pregnancy. They were then introduced to the PROMISES study and salivary progesterone as a screening tool for PTB. All interviews with ASHAs and pregnant women were conducted in primary health centres.

RECRUITMENT & DATA COLLECTION

All the participants provided their written informed consent.

Pre-post training questionnaire – ASHAs

For peer review only

A questionnaire of 25 items was designed to collect information on ASHAs expertise, employment, work experience and general knowledge of PTB and neonatal care. The questionnaire was administered to 201 ASHAs recruited in the PROMISES study before and after training within both districts (n=112 from Panna, n=89 from Satna).

Cross-sectional questionnaire – Women

Survey of the first 400 pregnant women participating in PROMISES and providing a saliva sample exploring women's experiences of salivary progesterone testing and its acceptability by the women.

Interviews – Women & ASHAs

Through opportunity sampling, ASHAs (n=10) and pregnant women (n=9, PROMISES study participants) were interviewed by the local research team. Interviews were face-to-face, structured and informational, exploring the health services provided to pregnant women, the role of ASHAs in caring for pregnant women and managing PTB, and women's thoughts on PTB and their experiences of salivary progesterone testing including feasibility and any challenges of this test within the rural setting. Interviews were audio-recorded (with the participants' consent), transcribed verbatim and translated into English.

PATIENT AND PUBLIC INVOLVEMENT

We did not directly include PPI, but the study rationale was presented and discussed with our UK NIHR recognised Preterm birth PPI group and the acceptability of the research was informed by participant feedback from a similar UK based study. Due to the participatory nature of this research, this study provides important PPI from women and community healthcare workers in rural India who are rarely consulted about research.

DATA ANALYSIS

Descriptive analysis of questionnaire data was conducted in IBM SPSS version 24 with results expressed as counts and percentages. Knowledge before and after

For peer review only

training was compared by the McNemar Test and a value of 0.05 considered to indicate statistical difference.

Preliminary analysis of the 19 interviews was undertaken by a member of the main PROMISES team [PS] with further detailed analysis by two other study team members [SAS, DCA]. Codes and generated themes were cross-checked with members of the wider PROMISES team. A thematic framework analysis was employed (Spencer, Ritchie et al. 2014) focussing on the perceptions of the intervention, the influencing factors which affected women's engagement with the service; and the function and implementation of the intervention. The framework was devised and agreed [SAS, DCA] using the overarching research questions, the interview schedules, and notes made when familiarising with the interview transcripts.

In-line with a multi-disciplinary approach to thematic framework analysis (Gale, Heath et al. 2013), interviews were first read in their entirety by three analysts to achieve familiarity with the interview content. One of the analysts had undertaken some of the interviews [PS], whilst the other two had not been involved in the data collection [DCA, SAS]. Interviews were then uploaded into NVivo for data management and analysis. Coding was focused using the framework to guide analysis. Theme names within the framework were adapted and refined as the analytical processes went on. Analysis was completed in a way which met both public and global health needs whilst being sensitive to the socio-economic and cultural environment in which these participants resided (Pope, Ziebland et al. 2000, Squires 2008, Squires 2009). Theme saturation was reached after analysis of approximately 60% of transcripts had been analysed (Vasileiou, Barnett et al. 2018) , which although achieved with relatively few transcripts, is unsurprising given the structured nature of the interviews (Francis, Johnston et al. 2010). For each of the main themes, data were charted, and matrices used to analyse patterns, sorting by participant status (i.e. ASHAs or women) and divided into barriers and facilitators. The most eloquent example quotations have been selected to represent the themes and analysis.

RESULTS

For peer review only

Two hundred and one ASHA's completed the training programme. Table 1 shows the geographic and demographic characteristics of ASHA's undertaking the training programme, of the 201 ASHA's 56% were from the Panna district and 44% from Satna district. Although there was a range in experience levels of the ASHAs, on average they had a high level of education and advanced experience.

<Insert Table 1 Here>

Pre-post training programme questionnaire – ASHAs

Before the one day training programme, approximately three quarters of the ASHAs had heard of PTB, however their knowledge of the risk factors, causes and postnatal support required for PTB was very limited (Table 2). After the training programme, there was a significant improvement in the ASHAs' knowledge of PTB compared with before training, with a 21% increase in the proportion of ASHAs that had heard of PTB, 49% increase in knowledge that PTB can cause newborn death, 52% increase in additional postnatal support required after PTB and 37% increase in correct knowledge of PTB risk factors. Before the training programme none of the 201 ASHAs were aware of potential tests for PTB, this is compared with 61% after training.

<Insert Table 2 Here>

Over half of the ASHAs reported they had experience of caring for women with PTB (Table 3), however their experience of providing women with information on PTB showed that over a quarter of the women are not interested in the information provided and over 90% of those who answered this question said family members were not interested in the information.

<Insert Table 3 Here>

Cross-sectional questionnaire – Pregnant women

Table 4 shows the geographic and demographic characteristics of the first four hundred women to provide a saliva sample within the PROMISES cohort. The average age was 24 years of age with 8 years of education.

<Insert Table 4 Here>

Women recruited to the PROMISES prospective cohort completed sections of a PROMISES questionnaire which captured information on women's socio-demographic and lifestyle characteristics, reproductive history, and saliva sampling. Within this questionnaire, women were asked three questions about the acceptability of the salivary test. These three questions have been extracted for the first 400 participants, of the 2000 cohort (Table 5). Despite all 400 women reporting the salivary test was acceptable and 82% finding the test easy, 84% reported the test was not better than drawing blood and 98% did not think the test was quick. What women disliked about the test was not clear as only three women reported they disliked associated mouth dryness, gagging or any embarrassment.

<Insert Table 5 Here>

Interviews – Pregnant women & ASHAs

Following analysis of interview data with ASHAs and women, our thematic framework comprised of three main areas: Implementation of Intervention; Networks of Influence; and Access to Healthcare (Tables 6 & 7). Contextual data was also captured as were details of local or traditional practices of, and attitudes towards, medicine, PTB and pregnancy ailments. This was not used for analysis, but instead for information to set the scene in which the data was collected.

The first area, Implementation of Intervention, covered information about women's perceived acceptability (degree of tolerance) of the intervention, as well as the feasibility and usefulness. The second area, Networks of Influence, was made up of themes of local knowledge, family and healthcare professional influences, and wider support networks. These forms of knowledge were noted as being transferred from the influencers and networks to women. Access to Healthcare comprised distance and cost of travel, transport issues as well as geographical factors.

<Insert Table 6 Here>

Implementation of Intervention

For peer review only

Implementation of Intervention, included two main subthemes: women’s perceived acceptability (degree of tolerance) towards the potential intervention, and the usefulness and feasibility. The intervention was perceived as acceptable by both ASHAs and pregnant women with 50% of ASHAs and 78% of women stating facilitating factors. This was compared to approximately 10% of both ASHAs and women who raised issues which suggested the intervention was not acceptable. No ASHAs or women raised issues in relation to the usefulness and feasibility of the intervention, however 20% of ASHAs and 44% of women highlighted factors which could be codified as useful and feasible.

Networks of Influence

Influencing factors was the largest theme and contained four main subthemes: healthcare professional influences, family influences, wider support networks, and local knowledge. Healthcare professional influence was mainly perceived as a transfer of knowledge from ASHAs and other healthcare professionals to pregnant women and was seen to be facilitatory in nature by women (78%) but not necessarily by ASHAs (20%). Despite this, only 10% of ASHAs and 22% of women suggested healthcare related knowledge transfer barriers. The next subtheme was the family influence. Within this subtheme 10% of ASHAs and approximately 30% of women suggested these to be facilitatory with 50% of ASHAs and 22% of women suggesting family influence acting as a barrier to their care. The third subtheme was wider support networks which was never seen as a facilitator. Analysis showed this subtheme was only raised by women (approximately 30%) as a barrier influencing their care. A final subtheme of local knowledge was seen by only ASHAs to pose some barriers to pregnant women accessing healthcare. In this respect 30% of ASHAs confirmed these barriers, whereas 20% of ASHAs and 22% of women considered local knowledge as a facilitator instead.

<Insert Table 7 Here>

Access to Healthcare

Access to Healthcare was made up of three subthemes: distance and cost of travel; mode of transport; and geographical factors.

For peer review only

Clearly distance was only ever seen as a barrier to accessing healthcare, with 20% of ASHAs and approximately 30% of women raising it as an issue. Furthermore transport was seen as a barrier to accessing healthcare by 30% of ASHAs and 11% of women. Similar numbers (10% of ASHAs and 22% of women) found transport which could meet their needs (though this may be a product of the study whereby ASHAs were offering transport as part of the study involvement). Finally, geographical factors were only occasionally mentioned, these were exclusively raised as barriers by women (22%).

DISCUSSION

This study presents an analysis of the feasibility and acceptability of using salivary based tests for predicting PTB, and the training of community health workers to facilitate the sampling. It has successfully demonstrated support for the research questions posed. A major strength of this study was the mixed methods approach implemented, whereby we were able to triangulate quantitative and qualitative data from both women and ASHAs. Mixed methods data triangulation can be undertaken in a number of different ways, and this study employed a parallel data analysis plan (Farmer, Robinson et al. 2006) . Parallel data analysis is where collection and analysis of both quantitative and qualitative datasets are carried out separately, and findings are not compared until the interpretation stage (Ostlund, Kidd et al. 2011, Creswell 2013).

In terms of health care worker training, it was clear that although ASHAs understood the concept of PTB (57% had experience of caring for women with PTB), their baseline knowledge of the condition was limited despite over 80% of ASHAs reporting five or more years of work experience and the standard government-issued training which is provided to all ASHAs (Ministry of Health & Family Welfare 2013). ASHAs understood PTB was a cause of neonatal death, but did not have clarity over the gestation cut off as a definition for PTB. Results of the questionnaire showed a lack of understanding by ASHAs of antenatal risk factors, antenatal tests, and postnatal support for PTB and preterm babies. This was further evidenced in both interview data collected from ASHAs. This is unsurprising as although community health workers have become a

For peer review only

more integral part of the health workforce in LMICs (Nebeker and López-Arenas 2016), basic health literacy remains low (El-Khayat 2017). In spite of this, previous literature has suggested that although health literacy may be universally poor, the work of community health workers is vitally important for hard to reach groups or those with high levels of social complexity (Uribe Guajardo, Slewa-Younan et al. 2018, Edmond, Yousufi et al. 2019, Saeedzai, Sadaat et al. 2019).

The one-day training sessions enabled ASHAs to have a greater understanding of PTB. As training evaluation was conducted directly after the training session, this only provides evidence of short term recall. Follow up of retention of information learnt and ability to apply knowledge would have been useful, though fell outside of the remit of this study. However, ASHAs were involved in the PROMISES study for approximately 24 months with continued contact with study field staff and pregnant women. As ASHAs played a key role in explaining the PROMISES study to women during the consent process, it is likely that knowledge was retained and that involvement in the study had a longer lasting impact on their knowledge of PTB as has been found in similar studies with community health workers who are engaged in research on pregnancy and/or women's health (Andrews, Felton et al. 2004, LeMasters, Baber Wallis et al. 2019).

A key goal of the study was to determine, in advance of potentially validating a saliva progesterone as a predictor of PTB for women in rural India, the acceptability of the test within this setting. Saliva sampling, whilst not as invasive a blood sampling, has been disliked by women in the UK for various reasons due to embarrassment and dryness of mouth (Carter, Cate et al. 2013). Reassuringly, both quantitative and qualitative data from women and ASHAs in the PROMISES study, shows the saliva progesterone testing to be acceptable. This is useful information, not only for our proposed progesterone test, but also for any other future point-of-care biomarker test.

Furthermore, although we did not formally measure health literacy in women participating in the PROMISES study, previous studies have shown that women's involvement in health-related research can promote better maternal health literacy (Johri, Subramanian et al. 2015, Barnes, Barclay et al. 2019), which may be a more holistic, if not anticipated, benefit of this study.

For peer review only - http://bmjopen.bmj.com/site/about/guidelines.xhtml

Strengths and Limitations:

A major strength of this study is the fact we present a mixed methods approach to understanding the acceptability of a novel intervention into a hard to reach (rural; socio-economically deprived) population in a low and middle-income country (LMIC) using community health workers. Mixed qualitative and quantitative methodological approaches have been hailed as providing the most viable way to identify and answer clinically relevant research questions (Shneerson and Gale 2015). Furthermore, the study presents data supporting the introduction of a previously un-used point of care test which can easily and quickly be utilised outside of formal healthcare settings, making this an important advance in prenatal care for rural populations in LMICs who may not have access to transport to services or to the healthcare facilities themselves (due to financial constraints, poor weather or infrastructure, or family influence). However, like all feasibility studies, we have identified certain limitations. Most notably, the qualitative data collected was assessed using (Morse 2000) as being of poor quality due to the high level of shadow data (i.e. participants talking about other people's experiences). We compensate for this by using a rigorous thematic framework methodological approach which enabled us to interrogate the data and distil it into coherent themes, whilst providing a percentage cover of all the topics raised in our framework for both women and ASHAs. Additionally, the opportunity sample could be seen as a methodological weakness. On consideration, these limitations are outweighed by the strengths of a study designed in a setting low in resources to demonstrate how simple interventions provide the possibility to improve the prenatal care of women and their babies.

Conclusion and future work:

In summary, these data provide support for our ongoing study of the feasibility of using salivary progesterone testing for prediction of preterm birth in rural settings. In addition, it highlights the potential usefulness of saliva for additional biomarker developments. The study further provides good evidence for this type of test being useful to shift cultural narratives to allow women more agency during pregnancy, however there is still a need for women to have better access to routine antenatal care even if these simple interventions can be carried out outside of formal healthcare settings. Although this study provides evidence for sample collection in homes and

For peer review only

then be returned to central facilities for testing in addition to successful mechanisms for reporting results; ideally this test should be undertaken in conjunction with routine antenatal care at healthcare facilities and so women should be afforded the time and opportunity to safely access these services.

LIST OF ABBREVIATION

Preterm birth (PTB)
Accredited Social Health Activist (ASHAs)
primary health care centres (PHCs)
low and middle-income country (LMIC)

REFERENCES

1. Andrews, J. O., G. Felton, M. E. Wewers and J. Heath (2004). "Use of Community Health Workers in Research With Ethnic Minority Women." Journal of Nursing Scholarship **36**(4): 358-365.
2. Barnes, L. A. J., L. Barclay, K. McCaffery and P. Aslani (2019). "Complementary medicine products: Information sources, perceived benefits and maternal health literacy." Women Birth **32**(6): 493-520.
3. Carter, J., R. Cate, A. Briley and L. Poston (2013). "PL.55 Testing Saliva for the Prediction of Preterm Birth: How Acceptable is This Method to Women at Risk?" Archives of Disease in Childhood - Fetal and Neonatal Edition **98**(Suppl 1): A69-A70.
4. Creswell, J. W. (2013). Research design: Qualitative, quantitative, and mixed methods approaches. Thousand Oaks, CA, Sage Publications, Incorporated.
5. Edmond, K., K. Yousufi, M. Naziri, A. Higgins-Steele, A. Q. Qadir, S. M. Sadat, A. L. Bellows and E. Smith (2019). "Mobile outreach health services for mothers and children in conflict-affected and remote areas: a population-based study from Afghanistan." Archives of Disease in Childhood: archdischild-2019-316802.
6. El-Khayat, Y. M. (2017). "A New Approach to Health Literacy: Working with Spanish Speaking Community Health Workers." Medical Reference Services Quarterly **36**(1): 32-41.
7. Farmer, T., K. Robinson, S. J. Elliott and J. Eyles (2006). "Developing and implementing a triangulation protocol for qualitative health research." Qualitative health research **16**(3): 377-394.

For peer review only

8. Fathima, F. N., M. Raju, K. S. Varadharajan, A. Krishnamurthy, S. R. Ananthkumar and P. K. Mony (2015). "Assessment of 'accredited social health activists'-a national community health volunteer scheme in Karnataka State, India." J Health Popul Nutr **33**(1): 137-145.
9. Francis, J. J., M. Johnston, C. Robertson, L. Glidewell, V. Entwistle, M. P. Eccles and J. M. Grimshaw (2010). "What is an adequate sample size? Operationalising data saturation for theory-based interview studies." Psychol Health **25**(10): 1229-1245.
10. Gale, N. K., G. Heath, E. Cameron, S. Rashid and S. Redwood (2013). "Using the framework method for the analysis of qualitative data in multi-disciplinary health research." BMC Med Res Methodol **13**: 117.
11. Johri, M., S. V. Subramanian, M. P. Sylvestre, S. Dudeja, D. Chandra, G. K. Kone, J. K. Sharma and S. Pahwa (2015). "Association between maternal health literacy and child vaccination in India: a cross-sectional study." J Epidemiol Community Health **69**(9): 849-857.
12. LeMasters, K., A. Baber Wallis, R. Chereches, M. Gichane, C. Tehei, A. Varga and K. Tumlinson (2019). "Pregnancy experiences of women in rural Romania: understanding ethnic and socioeconomic disparities." Culture, Health & Sexuality **21**(3): 249-262.
13. Liu, L., H. L. Johnson, S. Cousens, J. Perin, S. Scott, J. E. Lawn, I. Rudan, H. Campbell, R. Cibulskis, M. Li, C. Mathers and R. E. Black (2012). "Global, regional, and national causes of child mortality: an updated systematic analysis for 2010 with time trends since 2000." The Lancet **379**(9832): 2151-2161.
14. Liu, L., S. Oza, D. Hogan, J. Perin, I. Rudan, J. E. Lawn, S. Cousens, C. Mathers and R. E. Black (2015). "Global, regional, and national causes of child mortality in 2000-13, with projections to inform post-2015 priorities: an updated systematic analysis." Lancet **385**(9966): 430-440.
15. Ministry of Health & Family Welfare (2013). Induction Training Module for ASHAs.
16. Ministry of Health & Family Welfare (2013). NATIONAL RURAL HEALTH MISSION Meeting people's health needs in rural areas Framework for Implementation 2005-2012
17. Ministry of Health & Family Welfare (2017-2018). Annual Report of Department of Health and Family Welfare 2017-18.
18. Ministry of Home Affairs (2013). Sample Registration System (SRS)
19. Ministry of Home Affairs (2014). Annual Health Survey 2012 - 13 Fact Sheet: Madhya Pradesh.
20. Morse, J. M. (2000). "Determining Sample Size." Qualitative Health Research **10**(1): 3-5.
21. Nebeker, C. and A. López-Arenas (2016). "Building Research Integrity and Capacity (BRIC): An Educational Initiative to Increase Research Literacy among Community Health Workers and Promotores." Journal of microbiology & biology education **17**(1): 41-45.

For peer review only

22. Ostlund, U., L. Kidd, Y. Wengstrom and N. Rowa-Dewar (2011). "Combining qualitative and quantitative research within mixed method research designs: a methodological review." Int J Nurs Stud **48**(3): 369-383.
23. Pope, C., S. Ziebland and N. Mays (2000). "Qualitative research in health care. Analysing qualitative data." Bmj **320**(7227): 114-116.
24. Saeedzai, S. A., I. Sadaat, Z. Anwari, S. Hemat, S. Hadad, K. Osaki, M. Asaba, Y. Ishiguro, R. Mudassir, J. M. Burke, A. Higgins-Steele, K. Yousufi and K. M. Edmond (2019). "Home-based records for poor mothers and children in Afghanistan, a cross sectional population based study." BMC Public Health **19**(1): 766.
25. Sharma, P., S. Khan, M. Ghule, V. B. Shivkumar, R. Dargan, P. T. Seed, A. Sarkar, S. Mehra, P. V. Shivkumar and R. M. Tribe (2018). "Rationale & design of the PROMISES study: a prospective assessment and validation study of salivary progesterone as a test for preterm birth in pregnant women from rural India." Reprod Health **15**(1): 215.
26. Shneerson, C. L. and N. K. Gale (2015). "Using mixed methods to identify and answer clinically relevant research questions." Qual Health Res **25**(6): 845-856.
27. Spencer, L., J. Ritchie and W. O'Connor (2014). Analysis: practices, principles and processes' in Qualitative research practice: a guide for social science students and researchers. Qualitative research practice: a guide for social science students and researchers. J. Ritchie, J. Lewis, C. McNaughton Nicholls and R. Ormston. London, SAGE: 199-218.
28. Squires, A. (2008). "Language barriers and qualitative nursing research: methodological considerations." Int Nurs Rev **55**(3): 265-273.
29. Squires, A. (2009). "Methodological challenges in cross-language qualitative research: a research review." Int J Nurs Stud **46**(2): 277-287.
30. The Lancet (2012). "A timely arrival for Born Too Soon." Lancet **380**(9855): 1713.
31. Tripathy, P., N. Nair, R. Sinha, S. Rath, R. K. Gope, S. Rath, S. S. Roy, A. Bajpai, V. Singh, V. Nath, S. Ali, A. K. Kundu, D. Choudhury, S. K. Ghosh, S. Kumar, R. Mahapatra, A. Costello, E. Fottrell, T. A. Houweling and A. Prost (2016). "Effect of participatory women's groups facilitated by Accredited Social Health Activists on birth outcomes in rural eastern India: a cluster-randomised controlled trial." Lancet Glob Health **4**(2): e119-128.
32. Uribe Guajardo, M. G., S. Slewa-Younan, B. A. Kitchener, H. Mannan, Y. Mohammad and A. F. Jorm (2018). "Improving the capacity of community-based workers in Australia to provide initial assistance to Iraqi refugees with mental health problems: an uncontrolled evaluation of a Mental Health Literacy Course." International Journal of Mental Health Systems **12**(1): 2.
33. Vasileiou, K., J. Barnett, S. Thorpe and T. Young (2018). "Characterising and justifying sample size sufficiency in interview-based studies: systematic analysis of qualitative health research over a 15-year period." BMC Medical Research Methodology **18**(1): 148.

For peer review only

34. Vellakkal, S., H. Reddy, A. Gupta, A. Chandran, J. Fledderjohann and D. Stuckler (2017). "A qualitative study of factors impacting accessing of institutional delivery care in the context of India's cash incentive program." Soc Sci Med **178**: 55-65.
35. World Health Organization; March of Dimes; The Partnership for Maternal, N. C. H. S. t. C. (2012). Born too soon The global action report on preterm birth.

For peer review only

Table 1: Geographic and demographic characteristics of the ASHAs undertaking the preterm birth training programme.

Variable (n=201)	N	%
**District**		
**Panna**	112	55.7
**Satna**	89	44.3
**Years of education**		
**≤5 years**	7	3.5
**6-9 years**	68	33.8
**10-14 years**	110	54.7
**≥15 years**	16	8.0
**Mean (Standard deviation)**	10.15 (2.32)	
**Years of experience as an ASHA**		
**≤4 years**	37	18.4
**5-8 years**	70	34.8
**9-12 years**	94	46.8
**Mean (Standard deviation)**	7.50 (3.11)	

For peer review only

Table 2: ASHA's knowledge of preterm birth (PTB) before and after training programme

Question (n=201)	Before training		After training		P value
	N	%	N	%	
**PTB identified as a cause of newborn death**	97	48.3	196	97.5	0.000
**Had heard of PTB**	151	75.1	193	96.0	0.000
**Correctly defined PTB by gestation (< 37 weeks)**	49	24.4 (32.5*)	78	38.8 (40.2*)	0.253
**Correct knowledge of potential risk factors for PTB**	10	5.0 (6.6*)	84	41.8 (43.3*)	0.000
**Had any awareness of a test for PTB?**	0	0.0	122	60.7 (62.9)	.
**Correctly identified additional postnatal support needed for PTB**	31	15.4 (21.8*)	135	67.2 (69.6*)	0.000

*proportion with missing values excluded in the denominator

For peer review only

Table 3: ASHA's experiences of caring for women with preterm birth (PTB)

Question (n=201)	Before training	
	**N**	**%**
**Had dealt with PTB to date?**	114	56.7 (75.5*)
**What are your experiences (barriers) in providing information to women for preterm birth?**	47	23.4 (31.8*)
**Women are not interested**	135	67.2 (91.2*)
**Family members are not interested**	8	4.0 (5.4*)
**Other**		

*proportion with missing values excluded in the denominator

For peer review only

Table 4: Geographic and demographic characteristics of first 400 women to have a saliva sample recruited to PROMISES cohort

Variable / question (n=400)	N	%
**District**		
**Panna**	184	46.0
**Satna**	216	54.0
**Maternal age (years)**		
**18-20 years**	62	15.5
**21-25 years**	251	62.8
**26-30 years**	79	19.8
**>30**	8	2.0
**Mean (Standard deviation)**	23.6 (3.1)	
**Years of education**		
**≤4 years**	47	11.8
**5-8 years**	191	47.8
**9-12 years**	131	32.8
**>12 years**	31	7.8
**Mean (Standard deviation)**	7.8 (3.8)	
**Monthly income**[1]		
**Less than Rs. 2500**	261	62.3
**Rs. 2501-5000**	116	29.0
**Rs. 5001 to 7500**	12	3.0
**Rs. 7501 -10000**	7	1.8
**Rs. 10001 & above**	4	1.0
**Have you ever smoked or used tobacco in any form**		
**Yes and still use**	17	4.3
**Yes, gave up before pregnancy**	7	1.8
**No**	376	94.0
**Do you consume any drink containing alcohol?**		
**Yes**	1	0.3
**No**	399	99.8

[1] As of 2020 100 Indian Rupees = 1.41 US Dollars/1.08 Pound Sterling

For peer review only

Table 5: Acceptability of salivary progesterone test to the first 400 pregnant women recruited to the PROMISES cohort

Question (n=400)	N	%
**Did the participant find the salivary test acceptable?**		
**Yes**	400	100
**No**	0	0
**No opinion**	0	0
**Did the participant feel the salivary test was:**		
**Better than drawing blood?**		
**Yes**	65	16.3
**No**	335	83.8
**Easy to give?**		
**Yes**	325	81.3
**No**	75	18.8
**Quick?**		
**Yes**	9	2.3
**No**	391	97.8
**Convenient help to know the risks involved?**		
**Yes**	0	0
**No**	400	100
**None of the above**		
**Yes**	11	2.8
**No**	389	97.3
**Other**		
**Yes**	0	0
**No**	400	100
**What did the participant dislike about the salivary test:**		
**Mouth dryness?**		
**Yes**	7	1.8
**No**	393	98.3
**Time taken?**		
**Yes**	1	0.3
**No**	399	99.8
**Gag reflex?**		
**Yes**	2	0.5
**No**	398	99.5
**Embarrassment?**		
**Yes**	0	0
**No**	400	100
**None of the above?**		
**Yes**	391	97.8
**No**	9	2.3

For peer review only

**Other**		
**Yes**	0	0
**No**	400	100

For peer review only

Table 6: Thematic framework analysis of interviews with pregnant women

		Implementation of intervention	Networks of influence	Access to healthcare
Women	Facilitators	**Woman 4:** "I had no problem when I went for sonography. When I gave my sample, I asked outreach worker that why she needs my sample than she informed that they would examine it to determine the health of my child."<br><br>**Woman 8:** "I assume that women would agree to cooperate if they are told that it is for their child well-being. We, who have undergone this process can also convey and then I don't think women would hesitate."<br><br>**Woman 9:** "My saliva sample was collected at my house and the process was convenient to me."	**Woman 4:** "Outreach worker approached me along with ASHA and took me to district hospital. My husband stays in Panna and he comes to district hospital whenever I go for check-up."<br><br>**Woman 5:** "Usually it is either of my mother-in-law and sister-in-law accompanies me to sub-centre… I had no problem in travelling……… I got to know about preterm birth from outreach worker, she told me that baby who is born preterm is not healthy. It would be good to know more about pre-term birth."<br><br>**Woman 6:** "I will take care of my nutrition, as I do not want my child to born undernourished. Preterm birth can lead to under-nourishment of child. He remains weak. I have seen one example, there was a child who was born preterm and when he was growing, his legs were paralyzed."	**Woman 1:** "On my third visit, my sonography was conducted. As such, I did not face any difficulty......... we commuted by bus."<br><br>**Woman 8:** "My husband accompanies me to the health centres, so I face no problem in travelling. Public transport is also safe."
	Barrier	**Woman 7:** "Not all pregnant women would agree for providing their saliva, as many do not even go for sonography"	**Woman 7:** "They want to go [for sonography] but their families do not allow them. They do not have faith on government hospitals and say that they will go to the private hospitals for check-up."	**Woman 3:** "Travelling is problem during the rainy season as roads are not very good."<br><br>**Woman 6:** "I went for sonography twice, first I went to government hospital which is 20-25 km away and I waited for long time and doctor left for the day. Again, when I went to District hospital my sonography was not conducted. Then my in-laws suggested me to go to private hospital. Moreover, government hospitals reports are not reliable. So, I went to private hospital for my sonography."<br><br>**Woman 9:** "I faced no difficulties in saliva collection, but the ultrasound was a time taking procedure where I had to travel to a distance of more than 60 km and it took the whole day."

For peer review only

Table 7: Thematic framework analysis of interviews with ASHA's

		Implementation of intervention	Networks of influence	Access to healthcare
ASHA	Facilitators	**ASHA 3:** "Women are also very easily motivated for collecting saliva and for going for ultrasound."<br><br>**ASHA 8:** "Women are convinced to give saliva samples more as it is non-invasive and is for their benefit."	**ASHA 5:** "I accompany the project's outreach workers during saliva sample collection in order to motivate the pregnant women. Sometimes I accompany the pregnant women for ultrasound also as their families' requests to."<br><br>**ASHA 10:** "Sometimes I also [accompany] pregnant women for ultrasounds as their family members are more comfortable in sending their females with us rather than the outreach workers."	**ASHA 4:** "The women prefer going with me for ultrasound testing and are also able to commute by the auto rikshaws."
	Barrier	**ASHA 2:** "Since most women here are uneducated with high parity, so it is very difficult to make them understand. It is mostly their families who stop. They say what has to happen will happen, they already have 3-4 kids before as well."	**ASHA 1:** "It was easy to convince the pregnant women and family for ultrasound scan except in few cases where they were daily wagers and refused to go for ultrasound scan."<br><br>**ASHA 9:** "Pregnant women [are] willing to go for ultrasounds but sometimes family members refuse for this as they feel that it is not required."	**ASHA 1:** "The pregnant women deny [travel] for getting their ultrasound done as they are daily wagers."<br><br>**ASHA 2:** "Those women who are uneducated and poor they do not go for sonography. Even if tell them to go they would make some excuse. In government hospital if they go, most of the times it is closed and then these women do not go again……… One woman went for sonography, her sonography was not done that day, and now she refuses to go again."<br><br>**ASHA 5: "**Travelling is the major problem in going to primary healthcare centre for regular antenatal clinic. Many women also refuse due to lack of conveyance. Thus, many times I bring women on my own"

For peer review only

BMJ Open

The PROMISES Study: A mixed methods approach to explore the acceptability of salivary progesterone testing for pre-term birth risk amongst pregnant women and trained frontline healthcare workers in rural India.

Journal:	*BMJ Open*
Manuscript ID	bmjopen-2020-040268.R1
Article Type:	Original research
Date Submitted by the Author:	10-Nov-2020

Complete List of Authors:	Ashworth, Danielle; King's College London, Department of Women & Children's Health<br>Sharma, Pankhuri; MAMTA Health Institute for Mother and Child, Research and Innovation Unit<br>Silverio, Sergio; King's College London, Department for Women & Children's Health<br>Khan, Simi; MAMTA Health Institute for Mother and Child, Research and Innovation Unit<br>Kathuria, Nishtha; MAMTA Health Institute for Mother and Child, Research and Innovation Unit<br>Garg, Priyanka; MAMTA Health Institute for Mother and Child, Research and Innovation Unit<br>Ghule, Mohan; MAMTA Health Institute for Mother and Child, Research and Innovation Unit<br>Shivkumar, V; Mahatma Gandhi Institute of Medical Sciences, Department of Pathology<br>Tayade, Atul; Mahatma Gandhi Institute of Medical Sciences, Department of Radio-Diagnosis<br>Mehra, Sunil; MAMTA Health Institute for Mother and Child, Young People and Sexual and Reproductive Health and Rights Unit<br>Shivkumar, Poonam; Mahatma Gandhi Institute of Medical Sciences, Department of Obstetrics and Gynecology<br>Tribe, Rachel M.; King's College London, Department of Women & Children's Health

**Primary Subject Heading**:	Public health
Secondary Subject Heading:	Global health
Keywords:	PUBLIC HEALTH, Maternal medicine < OBSTETRICS, Prenatal diagnosis < OBSTETRICS

SCHOLARONE™
Manuscripts

I, the Submitting Author has the right to grant and does grant on behalf of all authors of the Work (as defined in the below author licence), an exclusive licence and/or a non-exclusive licence for contributions from authors who are: i) UK Crown employees; ii) where BMJ has agreed a CC-BY licence shall apply, and/or iii) in accordance with the terms applicable for US Federal Government officers or employees acting as part of their official duties; on a worldwide, perpetual, irrevocable, royalty-free basis to BMJ Publishing Group Ltd ("BMJ") its licensees and where the relevant Journal is co-owned by BMJ to the co-owners of the Journal, to publish the Work in this journal and any other BMJ products and to exploit all rights, as set out in our licence.

The Submitting Author accepts and understands that any supply made under these terms is made by BMJ to the Submitting Author unless you are acting as an employee on behalf of your employer or a postgraduate student of an affiliated institution which is paying any applicable article publishing charge ("APC") for Open Access articles. Where the Submitting Author wishes to make the Work available on an Open Access basis (and intends to pay the relevant APC), the terms of reuse of such Open Access shall be governed by a Creative Commons licence – details of these licences and which Creative Commons licence will apply to this Work are set out in our licence referred to above.

Other than as permitted in any relevant BMJ Author's Self Archiving Policies, I confirm this Work has not been accepted for publication elsewhere, is not being considered for publication elsewhere and does not duplicate material already published. I confirm all authors consent to publication of this Work and authorise the granting of this licence.

For peer review only

The PROMISES Study: A mixed methods approach to explore the acceptability of salivary progesterone testing for pre-term birth risk amongst pregnant women and trained frontline healthcare workers in rural India.

Danielle C. Ashworth,[1‡]
Pankhuri Sharma,[2‡]
Sergio A. Silverio,[1] ORCiD: 0000-0001-7177-3471
Simi Khan,[2]
Nishtha Kathuria,[2]
Priyanka R. Garg,[2]
Mohan D. Ghule,[2]
V.B. Shivkumar,[3]
Atul Tayade,[4]
Sunil Mehra,[5]
Poonam V. Shivkumar,[6]
Rachel M. Tribe (rachel.tribe@kcl.ac.uk),[1±] ORCiD: 0000-0003-3675-9978
and on behalf of the PROMISES Study Team.*

‡ Joint 1st Authors

± Corresponding Author: Professor Rachel Tribe, Dept. of Women and Children's Health, School of Life Course Sciences, King's College London, St Thomas' Hospital, London, SE1 7EH, United Kingdom, rachel.tribe@kcl.ac.uk, 0207 188 8241 (direct), 0207 188 3639 (admin)

*** The wider PROMISES Study Team includes:** Paul T. Seed (Department of Women & Children's Health, King's College London); Ritu Dargan (Obstetrics and Gynecology, MAMTA Health Institute for Mother and Child); and Archana Sarkar, Lalita Sengupta, Vikram Singh (Research and Innovation Unit, MAMTA Health Institute for Mother and Child).

For peer review only

[1] Department of Women & Children's Health, King's College London, London, UK
[2] Research and Innovation Unit, MAMTA Health Institute for Mother and Child, New Delhi, India
[3] Department of Pathology, Mahatma Gandhi Institute for Medical Sciences, Maharashtra, India
[4] Department of Radio-Diagnosis, Mahatma Gandhi Institute for Medical Sciences, Maharashtra, India
[5] Young People and Sexual and Reproductive Health and Rights Unit, MAMTA Health Institute for Mother and Child, New Delhi, India
[6] Department of Obstetrics and Gynecology, Mahatma Gandhi Institute for Medical Sciences, Maharashtra, India

Keywords: Preterm Birth, India, Salivary Progesterone, LMIC, Pregnancy

Word Count: 3,689

For peer review only

DECLARATIONS

Ethics approval and consent to participate

Ethical approval was obtained from both MAMTA Health Institute for Mother and Child (MAMTAHIMC) Ethical Review Board (MERB/Dec-2016/002) and the Institutional Review Board of Mahatma Gandhi Institute of Medical Science (MGIMS/IEC/OBGY/289/2016) prior to any recruitment. Written informed consent was obtained from pregnant women and ASHAs.

Consent for publication

Not applicable.

Data sharing statement

Data is not available to be shared.

Funding

This study was conducted as a part of project titled 'Low-cost salivary progesterone testing for detecting the risk of preterm births in rural community settings of India'. Funding for this project was provided by the Grand Challenge India, 2015 – All Children Thriving – India partners (Biotechnology Industry Research Assistance Council; Department of Biotechnology, India; and the Bill and Melinda Gates Foundation) for its overall implementation.

Authors' contributions

Conceptualization: [RMT, PVS, PS, SK, MDG, SM, and the wider PROMISES Study Team]; Methodology: [SAS, DCA, PS, SK, MDG, RMT]; Software: [SAS; DCA]; Validation: [RMT, PS]; Formal Analysis: [DCA, SAS, PS]; Investigation: [DCA, SAS]; Resources: [RMT, PVS, PS, SK, NK, PRG, MDG, VBS, AT, SM]; Data Curation: [PS, DCA, RMT, SAS]; Writing – Original Draft: [DCA, PS, SAS, RMT]; Writing – Review & Editing: [NK, PVS, PS, SK, NK, PRG, MDG, VBS, AT, SM, SAS, DCA, RMT]; Visualization: [SAS]; Supervision: [RMT, PVS, SM, MDG]; Project Administration: [RMT, PVS, PS, SK, NK, PRG, MDG, VBS, AT, SM]; Funding acquisition: [PVS, RMT, VBS, SM, and the wider PROMISES Study Team].

For peer review only

Acknowledgements

We would like to thank all the women who took part in this study, the ASHAs, and the clinical staff who performed the ultrasound scans.

Competing Interests

The authors declare that they have no competing interests.

Disclosure statement

Sergio A. Silverio (King's College London) is supported by the National Institute for Health Research Applied Research Collaboration South London [NIHR ARC South London] at King's College Hospital NHS Foundation Trust. The views expressed are those of the authors and not necessarily those of the NIHR or the Department of Health and Social Care.

Patient and Public Involvement

We did not directly include PPI, but the study rationale was presented and discussed with our UK NIHR recognised Preterm birth PPI group and the acceptability of the research was informed by participant feedback from a similar UK based study. Due to the participatory nature of this research, this study provides important PPI from women and community healthcare workers in rural India who are rarely consulted about research.

For peer review only

ABSTRACT

Introduction: India has an overall neonatal mortality rate of 28/1000 live births, with higher rates in rural India. Approximately 3.5 million pregnancies in India are affected by preterm birth annually and contribute to approximately a quarter of preterm births globally. Embedded within the PROMISES study (which aims to validate a low-cost salivary progesterone test for early detection of preterm birth risk) we present a mixed methods explanatory sequential feasibility sub-study of the salivary progesterone test.
Methods: A pre- and post-training questionnaire to assess Accredited Social Health Activists (n=201) knowledge and experience of preterm birth and salivary progesterone sampling was analysed using the McNemar test. Descriptive statistics for a cross-sectional survey of pregnant women (n=400) are presented in which the acceptability of this test for pregnant women is assessed. Structured interviews were undertaken with Accredited Social Health Activists (n=10) and pregnant women (n=9) and were analysed using Thematic Framework Analysis to explore the barriers and facilitators influencing the use of this test in rural India.
Results: Before training ASHAs knowledge of PTB (including risk factors, causes, postnatal support and testing) was very limited. After the training programme, there was a significant improvement in the ASHAs' knowledge of PTB. All four hundred women reported the salivary test was acceptable with the majority finding it easy but not quick or better than drawing blood. For the qualitative aspects of the study analysis of interview data with ASHAs and women, our thematic framework comprised of three main areas: Implementation of Intervention; Networks of Influence; and Access to Healthcare. Qualitative data were stratified and presented as barriers and facilitators.
Conclusion: This study suggests support for ongoing investigations validating preterm birth testing using salivary progesterone in rural settings.

For peer review only

ARTICLE SUMMARY: STRENGTHS AND LIMITATIONS OF THIS STUDY

- This is the first study to present a mixed methods approach to understanding the acceptability of salivary progesterone testing for risk of preterm birth in rural India.
- This study presents data supporting the introduction of a previously un-used point of care test which can easily and quickly be utilised outside of formal healthcare settings, and delivered by community healthcare workers.
- This study presents and integrates mixed methods data from both women and community healthcare workers in a rigorous and methodical way.
- This study was conducted in a low resource setting and demonstrates how a simple intervention can provide the possibility to improve the prenatal care of women and their babies.

For peer review only

INTRODUCTION

Globally, India's contribution to neonatal mortality is highest in the world with an estimate of 0.75 million [1] neonatal deaths in 2013. India's neonatal mortality rate is 28 per thousand live births, and in rural India the situation is even graver with 31 neonatal deaths per thousand live births [2]. Preterm birth (PTB) has been identified as one of the significant causes of neonatal deaths both in world [3] and in India [1]. Approximately 3.5 million pregnancies in India are affected by PTB annually [4-6], many infants die and those surviving often live with disability. Globally, PTBs are the highest in India (23.4% of all PTBs) [1, 7]. This burden is further affected by the low awareness level and utilization of health services, an issue that is exacerbated in rural India. Knowledge of, and around, PTB is limited even among community level health workers. This lack of knowledge hinders identification of mothers at risk of PTB and can delay access to health care services to manage such pregnancies. Considering the high burden of preterm birth and lack of knowledge thereof, an innovative study with an aim to validate a low-cost salivary progesterone (PROMISES) is being conducted for early detection of risk of PTB among pregnant women in two rural districts of India [8].

Even today, many myths and misconceptions prevail about pregnancy and childbirth in rural areas, mainly due to inadequate formal education, poor accessibility to healthcare services and lack of trained frontline health workers. Moreover, major role of decision making about health care of expectant mothers is mainly done by their immediate family members. The recent national survey in India indicates that only 11.4% women in rural India alone makes decision about their health care (NFHS-4). These contextual factors play a major role in implementing effective innovative interventions for improving maternal and neonatal health.

In rural India, government instituted community health care workers, Accredited Social Health Activist (ASHAs), play a significant role in linking pregnant women to maternal and child health care services [9]. These frontline health workers work in their residential communities/villages as health activists, educators and providers of basic essential services. ASHAs provide several support services to pregnant women [10] and invest considerable effort in identifying expectant and building trust with expectant

For peer review only

mothers and their family members. A multitude of evidence [11, 12] suggests that these links with frontline health workers have improved maternal and neonatal health interventions. For ASHA workers, the required minimum formal education [13] is only up to class 8th, hence it is important to provide adequate knowledge and regular trainings to ASHAs on topics of maternal and neonatal health in order to improve practice and sustainability of innovative interventions in these fields.

Hence, as part of the PROMISES study [8] two of the aims were: a) to assess the knowledge and educate the frontline health workers, ASHAs, on PTB and salivary progesterone sampling through a training programme; and b) to consider the feasibility, and acceptability to women and health workers, of using salivary progesterone in the rural settings for its further scale up as 'point of care test'. More specifically, the research objectives were to determine:

1. If training frontline health workers improved their knowledge on PTB and salivary progesterone sampling
2. Whether salivary progesterone PTB tests were acceptable to frontline health workers and pregnant women
3. The range of facilitating factors and barriers influencing the use of salivary progesterone PTB tests in Indian rural settings

METHODS

STUDY DESIGN & SETTING

A mixed methods study was undertaken to explore knowledge of PTB in frontline healthcare workers, and the acceptability and feasibility of salivary progesterone testing through a survey and structured interviews, respectively. This work formed part of a prospective study evaluating the feasibility and accuracy of saliva progesterone test to predict preterm birth, entitled – 'Low-cost salivary progesterone testing for detecting the risk of preterm births in rural community settings of India, PROMISES study' [8]. Participating healthcare workers were government instituted community health care workers, ASHAs, whose primary role is health education and promotion of good health practice and health service accessibility and utilization. ASHAs were

For peer review only

instrumental in recruitment of pregnant women for the PROMISES study, and thus capacity building through training was essential for effective implementation. Furthermore, the study explored the acceptability of salivary progesterone test among pregnant women eligible for PROMISES.

We conducted a pre-post training assessment of ASHAs knowledge of PTB; structured interviews with a sample of pregnant women and ASHAs discussing acceptability and feasibility of salivary progesterone testing for PTB; and a cross-sectional survey of pregnant women recruited to PROMISES which included questions on acceptability of salivary progesterone testing.

This study was carried out in the state of Madhya Pradesh, India, within two districts Panna and Satna, selected because of their high fertility and neonatal mortality rates [14, 15]. A one-day training programme was conducted for ASHAs at eight primary health care centres (PHCs). Overall four one-day training programmes were conducted, three at primary health centres and one at a district field office. Training included information about the maternal and child health services within the health systems, outreach services, high risk pregnancies, newborn care, PTBs, and general diagnostic services in pregnancy. They were then introduced to the PROMISES study and salivary progesterone as a screening tool for PTB. All interviews with ASHAs and pregnant women were conducted in primary health centres.

RECRUITMENT & DATA COLLECTION

All the participants provided their written informed consent. Both ASHA's and women were identified through their involvement in and eligibility for the main PROMISES study, details of inclusion/exclusion criteria can be found in the study protocol [8].

Pre-post training questionnaire – ASHAs

A questionnaire of 25 items was designed to collect information on ASHAs expertise, employment, work experience and general knowledge of PTB and neonatal care. The questionnaire was administered to 201 ASHAs recruited in the PROMISES study before and after training within both districts (n=112 from Panna, n=89 from Satna).

For peer review only

Cross-sectional questionnaire – Women

Survey of the first 400 pregnant women participating in PROMISES and providing a saliva sample exploring women's experiences of salivary progesterone testing and its acceptability by the women (n=184 from Panna, n=216 from Satna).

Interviews – Women & ASHAs

Through opportunity sampling, ASHAs (n=10, n=5 from Panna, n=5 from Satna) and pregnant women (n=9, n=5 from Panna, n=4 from Satna; one participant from the Satna region withdrew) were interviewed by two multilingual interviewers (authors PS, SK) who were part of the research team. Interviews were face-to-face, structured and informational using a topic guide developed using the research aims, the experiences of the research team and information received from the field team. Interviews explored the health services provided to pregnant women, the role of ASHAs in caring for pregnant women and managing PTB, and women's thoughts on PTB and their experiences of salivary progesterone testing including feasibility and any challenges of this test within the rural setting. Participants were offered travel compensation and refreshments as reimbursement for their time. Interviews were audio-recorded (with the participants' consent), transcribed verbatim and translated into English by PS and SK.

PATIENT AND PUBLIC INVOLVEMENT

We did not directly include PPI, but the study rationale was presented and discussed with our UK NIHR recognised Preterm birth PPI group and the acceptability of the research was informed by participant feedback from a similar UK based study. Due to the participatory nature of this research, this study provides important PPI from women and community healthcare workers in rural India who are rarely consulted about research.

DATA ANALYSIS

Quantitative data was uploaded and managed within the online secure database, MedSciNet. Descriptive analysis of questionnaire data was conducted in IBM SPSS version 24 with results expressed as counts and percentages. Knowledge before and

For peer review only

after training was compared by the McNemar Test and a value of 0.05 considered to indicate statistical difference.

Preliminary analysis of the 19 interviews was undertaken by a member of the main PROMISES team [PS] with further detailed analysis by two other study team members [SAS, DCA]. Codes and generated themes were cross-checked with members of the wider PROMISES team. A thematic framework analysis was employed [16] focussing on the perceptions of the intervention, the influencing factors which affected women's engagement with the service; and the function and implementation of the intervention. The framework was devised and agreed [SAS, DCA] using the overarching research questions, the interview schedules, and notes made when familiarising with the interview transcripts.

In-line with a multi-disciplinary approach to thematic framework analysis [17], interviews were first read in their entirety by three analysts to achieve familiarity with the interview content. One of the analysts had undertaken some of the interviews [PS], whilst the other two had not been involved in the data collection [DCA, SAS]. Interviews were then uploaded into NVivo for data management and analysis. Coding was focused using the framework to guide analysis. Theme names within the framework were adapted and refined as the analytical processes went on. Analysis was completed in a way which met both public and global health needs whilst being sensitive to the socio-economic and cultural environment in which these participants resided [18-20]. Theme saturation was reached after analysis of approximately 60% of transcripts had been analysed [21] , which although achieved with relatively few transcripts, is unsurprising given the structured nature of the interviews [22]. For each of the main themes, data were charted, and matrices used to analyse patterns, sorting by participant status (i.e. ASHAs or women) and divided into barriers and facilitators. The most eloquent example quotations have been selected to represent the themes and analysis.

RESULTS

For peer review only

Two hundred and one ASHA's completed the training programme. Table 1 shows the geographic and demographic characteristics of ASHA's undertaking the training programme, of the 201 ASHA's 56% were from the Panna district and 44% from Satna district. Although there was a range in experience levels of the ASHAs, on average they had a high level of education and advanced experience.

<Insert Table 1 Here>

Pre-post training programme questionnaire – ASHAs

Before the one day training programme, approximately three quarters of the ASHAs had heard of PTB, however their knowledge of the risk factors, causes and postnatal support required for PTB was very limited (Table 2). After the training programme, there was a significant improvement in the ASHAs' knowledge of PTB compared with before training, with a 21% increase in the proportion of ASHAs that had heard of PTB, 49% increase in knowledge that PTB can cause newborn death, 52% increase in additional postnatal support required after PTB and 37% increase in correct knowledge of PTB risk factors. Before the training programme none of the 201 ASHAs were aware of potential tests for PTB, this is compared with 61% after training.

<Insert Table 2 Here>

Over half of the ASHAs reported they had experience of caring for women with PTB (Table 3), however their experience of providing women with information on PTB showed that over a quarter of the women are not interested in the information provided and over 90% of those who answered this question said family members were not interested in the information.

<Insert Table 3 Here>

Cross-sectional questionnaire – Pregnant women

Table 4 shows the geographic and demographic characteristics of the first four hundred women to provide a saliva sample within the PROMISES cohort. The average age was 24 years of age with 8 years of education.

For peer review only

<Insert Table 4 Here>

Women recruited to the PROMISES prospective cohort completed sections of a PROMISES questionnaire which captured information on women's socio-demographic and lifestyle characteristics, reproductive history, and saliva sampling. Within this questionnaire, women were asked three questions about the acceptability of the salivary test. These three questions have been extracted for the first 400 participants, of the 2000 cohort (Table 5). Despite all 400 women reporting the salivary test was acceptable and 82% finding the test easy, 84% reported the test was not better than drawing blood and 98% did not think the test was quick. What women disliked about the test was not clear as only three women reported they disliked associated mouth dryness, gagging or any embarrassment.

<Insert Table 5 Here>

Interviews – Pregnant women & ASHAs

Following analysis of interview data with ASHAs and women, our thematic framework comprised of three main areas: Implementation of Intervention; Networks of Influence; and Access to Healthcare (Tables 6 & 7). Contextual data was also captured as were details of local or traditional practices of, and attitudes towards, medicine, PTB and pregnancy ailments. This was not used for analysis, but instead for information to set the scene in which the data was collected.

The first area, Implementation of Intervention, covered information about women's perceived acceptability (degree of tolerance) of the intervention, as well as the feasibility and usefulness. The second area, Networks of Influence, was made up of themes of local knowledge, family and healthcare professional influences, and wider support networks. These forms of knowledge were noted as being transferred from the influencers and networks to women. Access to Healthcare comprised distance and cost of travel, transport issues as well as geographical factors.

<Insert Table 6 Here>

Implementation of Intervention

For peer review only

Implementation of Intervention, included two main subthemes: women's perceived acceptability (degree of tolerance) towards the potential intervention, and the usefulness and feasibility. The intervention was perceived as acceptable by both ASHAs and pregnant women with 50% of ASHAs and 78% of women stating facilitating factors. This was compared to approximately 10% of both ASHAs and women who raised issues which suggested the intervention was not acceptable. No ASHAs or women raised issues in relation to the usefulness and feasibility of the intervention, however 20% of ASHAs and 44% of women highlighted factors which could be codified as useful and feasible.

Networks of Influence

Influencing factors was the largest theme and contained four main subthemes: healthcare professional influences, family influences, wider support networks, and local knowledge. Healthcare professional influence was mainly perceived as a transfer of knowledge from ASHAs and other healthcare professionals to pregnant women and was seen to be facilitatory in nature by women (78%) but not necessarily by ASHAs (20%). Despite this, only 10% of ASHAs and 22% of women suggested healthcare related knowledge transfer barriers. The next subtheme was the family influence. Within this subtheme 10% of ASHAs and approximately 30% of women suggested these to be facilitatory with 50% of ASHAs and 22% of women suggesting family influence acting as a barrier to their care. The third subtheme was wider support networks which was never seen as a facilitator. Analysis showed this subtheme was only raised by women (approximately 30%) as a barrier influencing their care. A final subtheme of local knowledge was seen by only ASHAs to pose some barriers to pregnant women accessing healthcare. In this respect 30% of ASHAs confirmed these barriers, whereas 20% of ASHAs and 22% of women considered local knowledge as a facilitator instead.

<Insert Table 7 Here>

Access to Healthcare

Access to Healthcare was made up of three subthemes: distance and cost of travel; mode of transport; and geographical factors.

For peer review only

Clearly distance was only ever seen as a barrier to accessing healthcare, with 20% of ASHAs and approximately 30% of women raising it as an issue. Furthermore transport was seen as a barrier to accessing healthcare by 30% of ASHAs and 11% of women. Similar numbers (10% of ASHAs and 22% of women) found transport which could meet their needs (though this may be a product of the study whereby ASHAs were offering transport as part of the study involvement). Finally, geographical factors were only occasionally mentioned, these were exclusively raised as barriers by women (22%).

DISCUSSION

This study presents an analysis of the feasibility and acceptability of using salivary based tests for predicting PTB, and the training of community health workers to facilitate the sampling. It has successfully demonstrated support for the research questions posed. A major strength of this study was the mixed methods approach implemented, whereby we were able to triangulate quantitative and qualitative data from both women and ASHAs. Mixed methods data triangulation can be undertaken in a number of different ways, and this study employed a parallel data analysis plan [23] . Parallel data analysis is where collection and analysis of both quantitative and qualitative datasets are carried out separately, and findings are not compared until the interpretation stage [24, 25].

In terms of health care worker training, it was clear that although ASHAs understood the concept of PTB (57% had experience of caring for women with PTB), their baseline knowledge of the condition was limited despite over 80% of ASHAs reporting five or more years of work experience and the standard government-issued training which is provided to all ASHAs [26]. ASHAs understood PTB was a cause of neonatal death, but did not have clarity over the gestation cut off as a definition for PTB. Results of the questionnaire showed a lack of understanding by ASHAs of antenatal risk factors, antenatal tests, and postnatal support for PTB and preterm babies. This was further evidenced in both interview data collected from ASHAs. This is unsurprising as although community health workers have become a more integral part of the health workforce in LMICs [27], basic health literacy remains low [28]. In spite of this,

For peer review only

previous literature has suggested that although health literacy may be universally poor, the work of community health workers is vitally important for hard to reach groups or those with high levels of social complexity [29-31].

The one-day training sessions enabled ASHAs to have a greater understanding of PTB. As training evaluation was conducted directly after the training session, this only provides evidence of short term recall. Follow up of retention of information learnt and ability to apply knowledge would have been useful, though fell outside of the remit of this study. However, ASHAs were involved in the PROMISES study for approximately 24 months with continued contact with study field staff and pregnant women. As ASHAs played a key role in explaining the PROMISES study to women during the consent process, it is likely that knowledge was retained and that involvement in the study had a longer lasting impact on their knowledge of PTB as has been found in similar studies with community health workers who are engaged in research on pregnancy and/or women's health [32, 33].

A key goal of the study was to determine, in advance of potentially validating a saliva progesterone as a predictor of PTB for women in rural India, the acceptability of the test within this setting. Saliva sampling, whilst not as invasive a blood sampling, has been disliked by women in the UK for various reasons due to embarrassment and dryness of mouth [34]. Reassuringly, both quantitative and qualitative data from women and ASHAs in the PROMISES study, shows the saliva progesterone testing to be acceptable. This is useful information, not only for our proposed progesterone test, but also for any other future point-of-care biomarker test.

Furthermore, although we did not formally measure health literacy in women participating in the PROMISES study, previous studies have shown that women's involvement in health-related research can promote better maternal health literacy [35, 36], which may be a more holistic, if not anticipated, benefit of this study.

Strengths and Limitations:

A major strength of this study is the fact we present a mixed methods approach to understanding the acceptability of a novel intervention into a hard to reach (rural; socio-economically deprived) population in a low and middle-income country (LMIC)

For peer review only

using community health workers. Mixed qualitative and quantitative methodological approaches have been hailed as providing the most viable way to identify and answer clinically relevant research questions [37]. Furthermore, the study presents data supporting the introduction of a previously un-used point of care test which can easily and quickly be utilised outside of formal healthcare settings, making this an important advance in prenatal care for rural populations in LMICs who may not have access to transport to services or to the healthcare facilities themselves (due to financial constraints, poor weather or infrastructure, or family influence). However, like all feasibility studies, we have identified certain limitations. Most notably, the qualitative data collected was assessed using [38] as being of poor quality due to the high level of shadow data (i.e. participants talking about other people's experiences). We compensate for this by using a rigorous thematic framework methodological approach which enabled us to interrogate the data and distil it into coherent themes, whilst providing a percentage cover of all the topics raised in our framework for both women and ASHAs. Additionally, the opportunity sample could be seen as a methodological weakness. Whilst the use of the qualitative data has helped maximise our understanding of the situation in rural India and helped to explain the quantitative findings, more could be done to enhance the generalisability of similar research in the future [39]. For example collecting relevant demographic information on each pregnancy (eg parity, multiple pregnancy, maternal age) may enable future qualitative analyses to stratify by these variables which may give more in depth insight. On consideration, these limitations are outweighed by the strengths of a study designed in a setting low in resources to demonstrate how simple interventions provide the possibility to improve the prenatal care of women and their babies.

Conclusion and future work:

In summary, these data provide support for our ongoing study of the feasibility of using salivary progesterone testing for prediction of preterm birth in rural settings. In addition, it highlights the potential usefulness of saliva for additional biomarker developments. The study further provides good evidence for this type of test being useful to shift cultural narratives to allow women more agency during pregnancy, however there is still a need for women to have better access to routine antenatal care even if these simple interventions can be carried out outside of formal healthcare settings. Although this study provides evidence for sample collection in homes and

For peer review only

then be returned to central facilities for testing in addition to successful mechanisms for reporting results; ideally this test should be undertaken in conjunction with routine antenatal care at healthcare facilities and so women should be afforded the time and opportunity to safely access these services.

LIST OF ABBREVIATION

Preterm birth (PTB)
Accredited Social Health Activist (ASHAs)
primary health care centres (PHCs)
low and middle-income country (LMIC)

REFERENCES

1. Liu, L., et al., *Global, regional, and national causes of child mortality in 2000-13, with projections to inform post-2015 priorities: an updated systematic analysis.* Lancet, 2015. **385**(9966): p. 430-40.
2. Ministry of Home Affairs, *Sample Registration System (SRS)* 2013.
3. Liu, L., et al., *Global, regional, and national causes of child mortality: an updated systematic analysis for 2010 with time trends since 2000.* The Lancet, 2012. **379**(9832): p. 2151-2161.
4. Blencowe, H., et al., *National, regional, and worldwide estimates of preterm birth rates in the year 2010 with time trends since 1990 for selected countries: a systematic analysis and implications.* Lancet, 2012. **379**(9832): p. 2162-72.
5. Chawanpaiboon, S., et al., *Global, regional, and national estimates of levels of preterm birth in 2014: a systematic review and modelling analysis.* Lancet Glob Health, 2019. **7**(1): p. e37-e46.
6. World Health Organization; March of Dimes; The Partnership for Maternal, N.C.H.S.t.C., *Born too soon The global action report on preterm birth*. 2012.
7. UNICEF, W., World Bank Group and United Nations, *Levels and trends in child mortality 2019. Estimates developed by the UN Inter-agency group for child mortality estimation*. 2019.
8. Sharma, P., et al., *Rationale & design of the PROMISES study: a prospective assessment and validation study of salivary progesterone as a test for preterm birth in pregnant women from rural India.* Reprod Health, 2018. **15**(1): p. 215.
9. Ministry of Health & Family Welfare, *Annual Report of Department of Health and Family Welfare 2017-18*. 2017-2018.
10. Vellakkal, S., et al., *A qualitative study of factors impacting accessing of institutional delivery care in the context of India's cash incentive program.* Soc Sci Med, 2017. **178**: p. 55-65.
11. Fathima, F.N., et al., *Assessment of 'accredited social health activists'-a national community health volunteer scheme in Karnataka State, India.* J Health Popul Nutr, 2015. **33**(1): p. 137-45.

For peer review only

12. Tripathy, P., et al., *Effect of participatory women's groups facilitated by Accredited Social Health Activists on birth outcomes in rural eastern India: a cluster-randomised controlled trial.* Lancet Glob Health, 2016. **4**(2): p. e119-28.
13. Ministry of Health & Family Welfare, *NATIONAL RURAL HEALTH MISSION Meeting people's health needs in rural areas Framework for Implementation 2005-2012* 2013.
14. The Lancet, *A timely arrival for Born Too Soon.* Lancet, 2012. **380**(9855): p. 1713.
15. Ministry of Home Affairs, *Annual Health Survey 2012 - 13 Fact Sheet: Madhya Pradesh*. 2014.
16. Spencer, L., J. Ritchie, and W. O'Connor, *Analysis: practices, principles and processes' in Qualitative research practice: a guide for social science students and researchers*, in *Qualitative research practice: a guide for social science students and researchers*, J. Ritchie, et al., Editors. 2014, SAGE: London. p. 199-218.
17. Gale, N.K., et al., *Using the framework method for the analysis of qualitative data in multi-disciplinary health research.* BMC Med Res Methodol, 2013. **13**: p. 117.
18. Pope, C., S. Ziebland, and N. Mays, *Qualitative research in health care. Analysing qualitative data.* Bmj, 2000. **320**(7227): p. 114-6.
19. Squires, A., *Language barriers and qualitative nursing research: methodological considerations.* Int Nurs Rev, 2008. **55**(3): p. 265-73.
20. Squires, A., *Methodological challenges in cross-language qualitative research: a research review.* Int J Nurs Stud, 2009. **46**(2): p. 277-87.
21. Vasileiou, K., et al., *Characterising and justifying sample size sufficiency in interview-based studies: systematic analysis of qualitative health research over a 15-year period.* BMC Medical Research Methodology, 2018. **18**(1): p. 148.
22. Francis, J.J., et al., *What is an adequate sample size? Operationalising data saturation for theory-based interview studies.* Psychol Health, 2010. **25**(10): p. 1229-45.
23. Farmer, T., et al., *Developing and implementing a triangulation protocol for qualitative health research.* Qualitative health research, 2006. **16**(3): p. 377-394.
24. Creswell, J.W., *Research design: Qualitative, quantitative, and mixed methods approaches.* 2013, Thousand Oaks, CA: Sage Publications, Incorporated.
25. Ostlund, U., et al., *Combining qualitative and quantitative research within mixed method research designs: a methodological review.* Int J Nurs Stud, 2011. **48**(3): p. 369-83.
26. Ministry of Health & Family Welfare, *Induction Training Module for ASHAs*. 2013.
27. Nebeker, C. and A. López-Arenas, *Building Research Integrity and Capacity (BRIC): An Educational Initiative to Increase Research Literacy among Community Health Workers and Promotores.* Journal of microbiology & biology education, 2016. **17**(1): p. 41-45.
28. El-Khayat, Y.M., *A New Approach to Health Literacy: Working with Spanish Speaking Community Health Workers.* Medical Reference Services Quarterly, 2017. **36**(1): p. 32-41.
29. Edmond, K., et al., *Mobile outreach health services for mothers and children in conflict-affected and remote areas: a population-based study from Afghanistan.* Archives of Disease in Childhood, 2019: p. archdischild-2019-316802.
30. Saeedzai, S.A., et al., *Home-based records for poor mothers and children in Afghanistan, a cross sectional population based study.* BMC Public Health, 2019. **19**(1): p. 766.
31. Uribe Guajardo, M.G., et al., *Improving the capacity of community-based workers in Australia to provide initial assistance to Iraqi refugees with mental health problems: an uncontrolled evaluation of a Mental Health Literacy Course.* International Journal of Mental Health Systems, 2018. **12**(1): p. 2.
32. Andrews, J.O., et al., *Use of Community Health Workers in Research With Ethnic Minority Women.* Journal of Nursing Scholarship, 2004. **36**(4): p. 358-365.
33. LeMasters, K., et al., *Pregnancy experiences of women in rural Romania: understanding ethnic and socioeconomic disparities.* Culture, Health & Sexuality, 2019. **21**(3): p. 249-262.

For peer review only

34. Carter, J., et al., *PL.55 Testing Saliva for the Prediction of Preterm Birth: How Acceptable is This Method to Women at Risk?* Archives of Disease in Childhood - Fetal and Neonatal Edition, 2013. **98**(Suppl 1): p. A69-A70.
35. Barnes, L.A.J., et al., *Complementary medicine products: Information sources, perceived benefits and maternal health literacy.* Women Birth, 2019. **32**(6): p. 493-520.
36. Johri, M., et al., *Association between maternal health literacy and child vaccination in India: a cross-sectional study.* J Epidemiol Community Health, 2015. **69**(9): p. 849-57.
37. Shneerson, C.L. and N.K. Gale, *Using mixed methods to identify and answer clinically relevant research questions.* Qual Health Res, 2015. **25**(6): p. 845-56.
38. Morse, J.M., *Determining Sample Size.* Qualitative Health Research, 2000. **10**(1): p. 3-5.
39. O'Cathain, A., et al., *Maximising the impact of qualitative research in feasibility studies for randomised controlled trials: guidance for researchers.* Pilot Feasibility Stud, 2015. **1**: p. 32.

For peer review only

Table 1: Geographic and demographic characteristics of the ASHAs undertaking the preterm birth training programme.

Variable (n=201)	N	%
**District**		
**Panna**	112	55.7
**Satna**	89	44.3
**Years of education**		
**≤5 years**	7	3.5
**6-9 years**	68	33.8
**10-14 years**	110	54.7
**≥15 years**	16	8.0
**Mean (Standard deviation)**	10.15 (2.32)	
**Years of experience as an ASHA**		
**≤4 years**	37	18.4
**5-8 years**	70	34.8
**9-12 years**	94	46.8
**Mean (Standard deviation)**	7.50 (3.11)	

For peer review only

Table 2: ASHA's knowledge of preterm birth (PTB) before and after training programme

Question (n=201)	Before training		After training		P value
	N	%	N	%	
**PTB identified as a cause of newborn death**	97	48.3	196	97.5	0.000
**Had heard of PTB**	151	75.1	193	96.0	0.000
**Correctly defined PTB by gestation (< 37 weeks)**	49	24.4 (32.5*)	78	38.8 (40.2*)	0.253
**Correct knowledge of potential risk factors for PTB**	10	5.0 (6.6*)	84	41.8 (43.3*)	0.000
**Had any awareness of a test for PTB?**	0	0.0	122	60.7 (62.9)	.
**Correctly identified additional postnatal support needed for PTB**	31	15.4 (21.8*)	135	67.2 (69.6*)	0.000

*proportion with missing values excluded in the denominator

For peer review only

Table 3: ASHA's experiences of caring for women with preterm birth (PTB)

Question (n=201)	Before training N	%
**Had dealt with PTB to date?**	114	56.7 (75.5*)
**What are your experiences (barriers) in providing information to women for preterm birth?**	47	23.4 (31.8*)
**Women are not interested**	135	67.2 (91.2*)
**Family members are not interested**	8	4.0 (5.4*)
**Other**		

*proportion with missing values excluded in the denominator

For peer review only

Table 4: Geographic and demographic characteristics of first 400 women to have a saliva sample recruited to PROMISES cohort

Variable / question (n=400)	N	%
**District**		
**Panna**	184	46.0
**Satna**	216	54.0
**Maternal age (years)**		
**18-20 years**	62	15.5
**21-25 years**	251	62.8
**26-30 years**	79	19.8
**>30**	8	2.0
**Mean (Standard deviation)**	23.6 (3.1)	
**Years of education**		
**≤4 years**	47	11.8
**5-8 years**	191	47.8
**9-12 years**	131	32.8
**>12 years**	31	7.8
**Mean (Standard deviation)**	7.8 (3.8)	
**Monthly income**[1]		
**Less than Rs. 2500**	261	62.3
**Rs. 2501-5000**	116	29.0
**Rs. 5001 to 7500**	12	3.0
**Rs. 7501 -10000**	7	1.8
**Rs. 10001 & above**	4	1.0
**Have you ever smoked or used tobacco in any form**		
**Yes and still use**	17	4.3
**Yes, gave up before pregnancy**	7	1.8
**No**	376	94.0
**Do you consume any drink containing alcohol?**		
**Yes**	1	0.3
**No**	399	99.8

[1] As of 2020 100 Indian Rupees = 1.41 US Dollars/1.08 Pound Sterling

For peer review only

Table 5: Acceptability of salivary progesterone test to the first 400 pregnant women recruited to the PROMISES cohort

Question (n=400)	N	%
**Did the participant find the salivary test acceptable?**		
**Yes**	400	100
**No**	0	0
**No opinion**	0	0
**Did the participant feel the salivary test was:**		
**Better than drawing blood?**		
**Yes**	65	16.3
**No**	335	83.8
**Easy to give?**		
**Yes**	325	81.3
**No**	75	18.8
**Quick?**		
**Yes**	9	2.3
**No**	391	97.8
**Convenient help to know the risks involved?**		
**Yes**	0	0
**No**	400	100
**None of the above**		
**Yes**	11	2.8
**No**	389	97.3
**Other**		
**Yes**	0	0
**No**	400	100
**What did the participant dislike about the salivary test:**		
**Mouth dryness?**		
**Yes**	7	1.8
**No**	393	98.3
**Time taken?**		
**Yes**	1	0.3
**No**	399	99.8
**Gag reflex?**		
**Yes**	2	0.5
**No**	398	99.5
**Embarrassment?**		
**Yes**	0	0
**No**	400	100
**None of the above?**		
**Yes**	391	97.8
**No**	9	2.3

For peer review only

**Other**		
**Yes**	0	0
**No**	400	100

For peer review only

Table 6: Thematic framework analysis of interviews with pregnant women

		Implementation of intervention	Networks of influence	Access to healthcare
Women	Facilitators	**Woman 4 (Panna):** "I had no problem when I went for sonography. When I gave my sample, I asked outreach worker that why she needs my sample than she informed that they would examine it to determine the health of my child."<br><br>**Woman 8 (Satna):** "I assume that women would agree to cooperate if they are told that it is for their child well-being. We, who have undergone this process can also convey and then I don't think women would hesitate."<br><br>**Woman 9 (Panna):** "My saliva sample was collected at my house and the process was convenient to me."	**Woman 4 (Panna):** "Outreach worker approached me along with ASHA and took me to district hospital. My husband stays in Panna and he comes to district hospital whenever I go for check-up."<br><br>**Woman 5 (Satna):** "Usually it is either of my mother-in-law and sister-in-law accompanies me to sub-centre… I had no problem in travelling……… I got to know about preterm birth from outreach worker, she told me that baby who is born preterm is not healthy. It would be good to know more about pre-term birth."<br><br>**Woman 6 (Satna):** "I will take care of my nutrition, as I do not want my child to born undernourished. Preterm birth can lead to under-nourishment of child. He remains weak. I have seen one example, there was a child who was born preterm and when he was growing, his legs were paralyzed."	**Woman 1 (Panna):** "On my third visit, my sonography was conducted. As such, I did not face any difficulty......... we commuted by bus."<br><br>**Woman 8 (Satna):** "My husband accompanies me to the health centres, so I face no problem in travelling. Public transport is also safe."
	Bar rier	**Woman 7 (Satna):** "Not all pregnant women would agree for providing their saliva, as many do not even go for sonography"	**Woman 7 (Satna):** "They want to go [for sonography] but their families do not allow them. They do not have faith on government hospitals and say that they will go to the private hospitals for check-up."	**Woman 3 (Panna):** "Travelling is problem during the rainy season as roads are not very good."<br><br>**Woman 6 (Satna):** "I went for sonography twice, first I went to government hospital which is 20-25 km away and I waited for long time and doctor left for the day. Again, when I went to District hospital my sonography was not conducted. Then my in-laws suggested me to go to private hospital. Moreover, government hospitals reports are not reliable. So, I went to private hospital for my sonography."<br><br>**Woman 9 (Panna):** "I faced no difficulties in saliva collection, but the ultrasound was a time taking

For peer review only

				procedure where I had to travel to a distance of more than 60 km and it took the whole day.”

Table 7: Thematic framework analysis of interviews with ASHA’s

		Implementation of intervention	Networks of influence	Access to healthcare
ASHA	Facilitators	**ASHA 3 (Panna):** “Women are also very easily motivated for collecting saliva and for going for ultrasound.”<br><br>**ASHA 8 (Satna):** “Women are convinced to give saliva samples more as it is non-invasive and is for their benefit.”	**ASHA 5 (Panna):** “I accompany the project’s outreach workers during saliva sample collection in order to motivate the pregnant women. Sometimes I accompany the pregnant women for ultrasound also as their families’ requests to.”<br><br>**ASHA 10 (Satna):** “Sometimes I also [accompany] pregnant women for ultrasounds as their family members are more comfortable in sending their females with us rather than the outreach workers.”	**ASHA 4 (Panna):** “The women prefer going with me for ultrasound testing and are also able to commute by the auto rikshaws.”
	Barrier	**ASHA 2 (Panna):** “Since most women here are uneducated with high parity, so it is very difficult to make them understand. It is mostly their families who stop. They say what has to happen will happen, they already have 3-4 kids before as well.”	**ASHA 1 (Panna):** “It was easy to convince the pregnant women and family for ultrasound scan except in few cases where they were daily wagers and refused to go for ultrasound scan.”<br><br>**ASHA 9 (Satna):** “Pregnant women [are] willing to go for ultrasounds but sometimes family members refuse for this as they feel that it is not required.”	**ASHA 1 (Panna):** “The pregnant women deny [travel] for getting their ultrasound done as they are daily wagers.”<br><br>**ASHA 2 (Panna):** “Those women who are uneducated and poor they do not go for sonography. Even if tell them to go they would make some excuse. In government hospital if they go, most of the times it is closed and then these women do not go again……… One woman went for sonography, her sonography was not done that day, and now she refuses to go again.”<br><br>**ASHA 5 (Panna): “**Travelling is the major problem in going to primary healthcare centre for regular antenatal clinic. Many women also refuse due to lack of conveyance. Thus, many times I bring women on my own”

For peer review only
